# Uncovering the geographical and host impacts on the classification of *Vibrio vulnificus*

**DOI:** 10.1111/eva.12602

**Published:** 2018-02-20

**Authors:** Shoukai Yu

**Affiliations:** ^1^ Program in Molecular and Integrative Physiological Sciences Department of Environmental Health Harvard T. H. Chan School of Public Health Boston MA USA

**Keywords:** bioinformatics/phyloinformatics, molecular evolution, phylogeography, virulence

## Abstract

*Vibrio vulnificus* causes human sickness throughout the world via the consumption of undercooked seafood or exposure to contaminated water. Previous attempts at phylogenetic analyses of *V. vulnificus* have proven unsuccessful, mainly due to the poorly understood impact of factors on its divergence. In this study, we used advanced statistical and phylogenetic methods to strengthen the classification of *V. vulnificus*. This updated classification included the impact of geographical and host factors. The results demonstrate the existence of hierarchies and multidimensional effects in the classification of *V. vulnificus*, from the molecular level using biotypes, to the distributional level using geographical location, to the adaptational level through host immune response. These findings have implications for the classification of bacteria, bacterial evolution, and public health.

## 1 INTRODUCTION

The pathogenic bacterium *Vibrio vulnificus* is Gram‐negative, curved, and motile. There are three main disease syndromes caused by this bacterium: invasive septicemia, acute gastroenteritis, and necrotizing wound infections. *Vibrio vulnificus* naturally exists in coastal regions globally and can cause seafood‐borne disease and wound infections in humans and a range of fish diseases. Although *V. vulnificus* can cause severe and even rare fatal infections in infected individuals, the impact of climate change and geographical distribution on the ecology of *V. vulnificus* is little known. A series of environmental parameters contribute to the virulence and abundance of *V. vulnificus*, which includes ocean temperature, turbidity, conductivity, sea level height, sea ice, winds, salinity, and wave length and velocity of propagation. The ecology of *V. vulnificus* is also related to dissolved organic carbon and chlorophyll (Johnson et al., 2012; Shaw, Jacobs, & Crump, 2014; Sterk et al., 2015). Both climate factors and host sources can affect the ecology and evolution of *V. vulnificus*. For example, a growing body of recent publications have reported correlations between climate change and the infection rate of *V. vulnificus* (Abraham et al., 2013; Baker‐Austin et al., 2013; Greer, Ng, & Fisman, 2008; Harvell et al., 1999; Hayes et al., 2001; Sterk et al., 2015), which could be directly linked to geographical factors. The global distribution of liver diseases could also be potentially linked to infections of *V. vulnificus* (Chuang, Yuan, Liu, Lan, & Huang, 1992; Wong, Liu, & Chen, 2005). Overall, geographical variation can represent many factors, such as climate and the distribution of host immune system responses. A great amount of effort has been put into trying to distinguish *V. vulnificus* strains by virulence or avirulence and to classify them by environmental and clinical strains (Amaro, Biosca, Fouz, Toranzo, & Garay, 1994; Krovacek, Baloda, Dumontet, & Månsson, 1994; Moreno, 1998; Morris et al., 1987; Rodrigues, Ribeiro, & Hofer, 1992; Stelma, Reyes, Peeler, Johnson, & Spaulding, 1992; Strom & Paranjpye, 2000; Tison & Kelly, 1986; Warner & Oliver, 1999). However, these efforts have largely proven unsuccessful (Phillips & Satchell, 2017). Moreover, none of these attempts have tried to incorporate geographical factors from currently available global databases into the analyses.

Although genomic and geographical data for *V. vulnificus* are publicly available and include isolates worldwide, studies on the effects of geographical isolation are still difficult to perform due to limitations of methodology and the multidisciplinary nature of the issue. In the current study, we integrated geographical factors and host attribution into an evolutionary analysis of *V. vulnificus*. Specifically, we used geographical locations and attributed sources of *V. vulnificus* (infected human samples, aquatic animal samples, and samples from the environment) to provide a better understanding of how to classify *V. vulnificus*. We estimated the divergence times of some specific strains of *V. vulnificus* and constructed a phylogeny. We also evaluated the distribution of genetic variation within and among defined groups and pairwise fixation indices (FST) were estimated. For each defined group (by geographical location or host source), the presence of population clusters was inferred by Bayesian analysis. The ultimate goal was to understand the interaction between the ecology of bacteria and their global distribution.

## 2 METHODS

### 2.1 Data sources

Four hundred and fifty‐two isolates of *V. vulnificus* were utilized in this analysis. The isolate data contain information on country, host, sampling year, sequence type (ST), and ten housekeeping genes via multilocus sequence typing (MLST) (PubMLST: http://pubmlst.org/). Neighbor‐joining trees were produced on all the isolates using MEGA 7 (Kumar, Stecher, & Tamura, 2016). The distance matrix of the mean number of pairwise differences was produced through Arlequin 3.5 (Excoffier & Lischer, 2010). Neighbor‐Net based on pairwise differences was constructed using the software SplitTree (Huson, 1998; Huson & Bryant, 2005). In a consensus tree, the agreed upon (compatible) part of a set of phylogenetic trees can be displayed as a splits‐graph (Holland, Delsuc, Moulton, & Baker, 2005). The incompatible parts can be represented by split networks of group trees.

Maximum likelihood (ML) is a general statistical method for identifying the topology that explains the evolution of observing the data (e.g., a set of aligned nucleotide sequences) under a given substitution model of evolution with the greatest likelihood (Felsenstein, 1981). In bioinformatics, neighbor joining (Saitou & Nei, 1987) is a bottom‐up clustering method that greedily optimizes the so‐called balanced minimum evolution criterion (Gascuel & Steel, 2006), and this algorithm requires knowledge of the distance between each pair of sequences to construct the phylogenetic trees, based on the given sequence data. Due to the efficiency for the analysis of large data sets, neighbor‐joining methods are wildly used for constructing phylogenetic trees from distance data (Gascuel & Steel, 2006).

### 2.2 Analysis of molecular variance

Analysis of molecular variance (AMOVA) was conducted to attribute the total variance into geographically related genetic structures or host‐associated genetic structures using Arlequin software (Excoffier & Lischer, 2010). As a hierarchical analysis of variance, AMOVA was performed in two ways in this study. For the first AMOVA, isolates from different geographical locations were separated into three groups: Asia, Europe, and United States, and then within each group, the isolates were divided by different host sources: human, aquatic animals, and the environment. For the second AMOVA, isolates from different host sources were separated into three groups: human, aquatic animals, and the environment; then, within each group, the isolates were divided by three geographical locations: Asia, Europe, and United States.

### 2.3 Analysis of population structure

STRUCTURE is a software package to use multilocus genotype data to investigate population structure. We used STRUCTURE to infer the presence of distinct populations that could be assigned to isolates (Falush, Stephens, & Pritchard, 2007; Pritchard, Wen, & Falush, 2010). There were nine sets (three sources multiplied by three locations) of data for 452 *V. vulnificus* isolates (Table S1). A no‐admixture model with selected options of USEPOPINFO, PopFlag, and PopData was applied in STRUCTURE (Hubisz, Falush, Stephens, & Pritchard, 2009; Pritchard, Stephens, & Donnelly, 2000). The no‐admixture model was employed with a burn‐in of 10,000 iterations. Convergence was assured by a comparison of multiple independent chains.

To eliminate the effects of different biotypes, 100 isolates were selected randomly from the original datasets of biotype 1 (294 isolates) and used as a training set. Host sources and geographical locations were prespecified for these 100 isolates in STRUCTURE (Table S2). Probabilistic assignment of alleles to different sources and different geographical locations were performed separately. These assignments were based on allele designations with 10,000 burn‐in iterations and with a subsequent 10,000 iterations. The assignment of 194 isolates was performed based on the training set of 100 separate isolates.

### 2.4 Bayesian phylogenetic analysis

The choice of isolates for Bayesian evolutionary analysis by sampling trees (BEAST) analysis was based on both geographical locations and host sources (Table 1), which meant nine possible combinations. Because the European isolates contained biotype 3 from Israel, and there is more ST variation from European countries, one additional isolate from Europe was also added in the analysis.

**Table 1:** The combination of selected sequence types for BEAST analysis

Source	Asian	European	USA
Environment	ST‐73	ST‐5	ST‐4
Human	ST‐77	ST‐8	ST‐3
Aquatic animals	ST‐158	ST‐138	ST‐30

Due to the higher diversity from European countries, one additional isolate (ST‐218, short for sequence type 218) was also added in the analysis.

The program BEAST (Drummond & Rambaut, 2007; Drummond, Suchard, Xie, & Rambaut, 2012) was used to estimate the divergence time for different STs of *V. vulnificus* and further to construct phylogenies of specific strains that represent different sources and geographical locations. The selected sequence types (STs) used are listed in Table 1. These STs were based on the ten loci characterized by MLST. The HKY substitution model was then applied in BEAST (Drummond & Rambaut, 2007; Drummond et al., 2012; Hasegawa, Kishino, & Yano, 1985). The iterations for Markov chain Monte Carlo (MCMC) were 100,000,000, and two MCMCs were compared to check the convergence. The posterior distribution had a large effective sample size (ESS) of 71,973; tree edge lengths were scaled.

## 3 RESULTS

### 3.1 Neighbor‐joining tree

**Figure 1:**
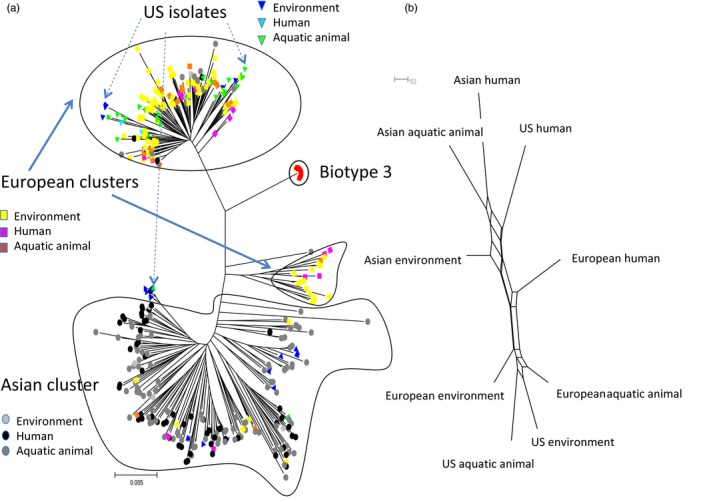
(a) Evolutionary history was inferred using the neighbor‐joining method (Saitou & Nei, 1987). The evolutionary distances were computed using the maximum composite likelihood method (Tamura, Nei, & Kumar, 2004) and are in units of the number of base substitutions per site. The analysis involved 452 nucleotide sequences. All positions containing gaps and missing data were eliminated. There were a total of 4,326 positions in the final dataset. Evolutionary analyses were conducted in MEGA7 (Kumar et al., 2016). Biotype 3 isolates are represented by red dots. Light gray dots represent isolates that are from Asian environmental sources. Black dots represent isolates that are from Asian human sources. Dark gray dots represent isolates that are from Asian aquatic animal sources. Yellow, pink, and orange represent isolates from European environment, human, and aquatic animal sources, respectively. Light blue, dark blue, and green represent isolates from US environment, human, and aquatic animal sources, respectively. (b) Neighbor‐net reconstruction of relationships among isolates from different geographical sampling locations and host sources using the software SplitTree

The mostly likely evolutionary tree of given taxon can be represented by a consensus tree. The consensus tree in Figure 1 shows the evolutionary history and phylogenetic relationships among isolates from three different geographical sampling locations and three host sources. The tree also demonstrates that the clusters of the three geographical locations transcend the host impacts.

### 3.2 Analysis of molecular variance

The amount of population genetic structure was evaluated by partitioning into total phenotypic variance within populations and among populations using the software Arlequin (v. 3.5) (Excoffier & Lischer, 2010). Pairwise FST indexes were calculated using genetic distances between populations. By definition, F statistics range from 0 to 1 (Holsinger & Weir, 2009). The value 0 for FST indicates no differentiation, while a value of 1 indicates complete differentiation.

For the AMOVAs, isolates from three geographical regions were separated into three groups and, within each group, the isolates were divided by different host sources: human, aquatic animals, and environment. Table 2 shows the results of AMOVAs when the geographical region grouping was set at a higher level. There is 21.2% variance assigned among geographical regions, only 18.2% variance assigned among hosts within each region, and the majority variance was assigned within each host for a fixed region. When AMOVAs were performed with the host sources set at a higher level and the geographical regions set at a lower level, there was no variance assigned among host sources. In summary, the effect of geographical regions was greater than the effect of host sources on the genetic variance of *V. vulnificus*. Furthermore, the majority of variance (about 60%) was assigned to within each geographical region and within each host source.

**Table 2:** AMOVA results with geographical regions defined as a higher grouping level, and then, the host sources defined as a lower level

Source of variation	Degrees of freedom	Sum of squares	Variance components
Among regions	2	4,541.3	11.9
Among hosts within regions	6	2,792	10.3
Within hosts	443	15,093.5	34.1

### 3.3 The phylogeny

**Figure 2:**
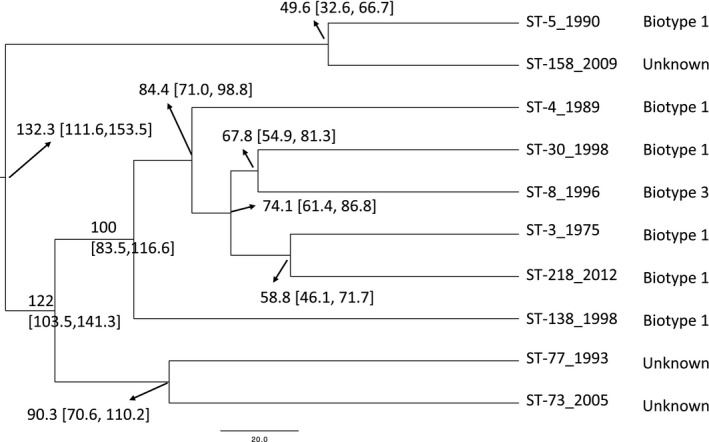
The phylogeny of ten selected isolates generated from Bayesian Evolutionary Analysis by Sampling Trees (BEAST) software. Mean divergence estimates are indicated at corresponding nodes in units of years before present. The 95% highest posterior density intervals for each node are given in brackets

From the BEAST analysis (Table 3 and Figure 2), the estimated divergence time of ST‐3 and ST‐5 is about 132 years ago (95% HPD: 111.6, 153.5). The highest posterior density (HPD) represents the smallest credible interval in the BEAST analyses that contains 95% of the posterior probability. In the topology (Figure 2), ST‐5 and ST‐158, ST‐30 and ST‐8, ST‐3 and ST‐218, ST‐77 and ST‐73 are grouped together, respectively. The estimated divergence time of ST‐5 and ST‐158 is about 50 years ago (95% HPD: 32.6 to 66.7, in Figure 2). The estimated divergence time of ST‐8 and ST‐30 is about 68 years ago (95% HPD: 54.9 to 81.3, in Figure 2). The estimated divergence time of ST‐3 and ST‐218 is about 60 years ago (95% HPD: 46.1 to 71.7, in Figure 2). The estimated divergence time of ST‐77 and ST‐73 is about 90 years ago (95% HPD: 70.6 to 110.2, in Figure 2).

**Table 3:** BEAST results of the mean split time

Splits Name	Time (unit year)	95% HPD lower, upper range
ST‐3_ST‐5	132.28	111.57, 153.49
ST‐3_ST‐73	121.99	103.48, 141.28
ST‐3_ST‐138	99.55	83.52, 116.53
ST‐3_ST‐4	84.38	70.98, 98.79
ST‐3_ST‐30	74.15	61.37, 86.85
ST‐73_ST‐77	90.35	70.63, 110.23
ST‐30_ST‐8	67.80	54.92, 81.29
ST‐3_ST‐218	58.78	46.10, 71.67
ST‐5_ST‐158	49.56	32.61, 66.69

HPD, highest posterior density; ST, sequence type.

### 3.4 Structure

For the source assignment (Table 4), the three host sources were predefined for 100 isolates, and then, the 194 isolates were assigned to each of the host sources using a no‐admixture model. Finally, the assignment results were compared to the true reported host sources to evaluate the robustness of each host source. For the geographical location assignment (Table 5), the three geographical locations were predefined for 100 isolates, and then, the 194 isolates were assigned to each. Finally, the assignment results were compared to the true reported geographical locations to evaluate the robustness of each host source. For both tables, on the diagonal from the upper left to lower right, the number represents isolates assigned to their own groups; the off‐diagonal numbers represent isolates assigned to other groups. Because there are more isolates assigned to their own group rather than the other two groups for geographical effects compared to host effects, the two above results show that the divergence effect created from geographical locations transcends the effect created by host association.

**Table 4:** The predicted host source of 294 biotype 1 isolates assigned to one of three host sources. Assignment of isolates to host sources was on the basis of STRUCTURE analysis, and the three host sources were defined using 100 isolates with predefined host sources

True sources	Cluster 1	Cluster 2	Cluster 3	Number of isolates
Environment	0.773	0.114	0.114	102
Human	0.076	0.739	0.186	76
Aquatic animals	0.172	0.191	0.637	116

**Table 5:** The predicted geographical locations of 294 biotype 1 isolates assigned to one of three sampling locations. Assignment of isolates to geographical locations was based on STRUCTURE analysis, and the three geographical locations were defined using 100 isolates with predefined true geographical locations of sampling sites

True locations	Cluster 1	Cluster 2	Cluster 3	Number of isolates
Asia	0.873	0.066	0.061	106
Europe	0.059	0.844	0.097	131
USA	0.169	0.138	0.693	57

**Figure 3:**
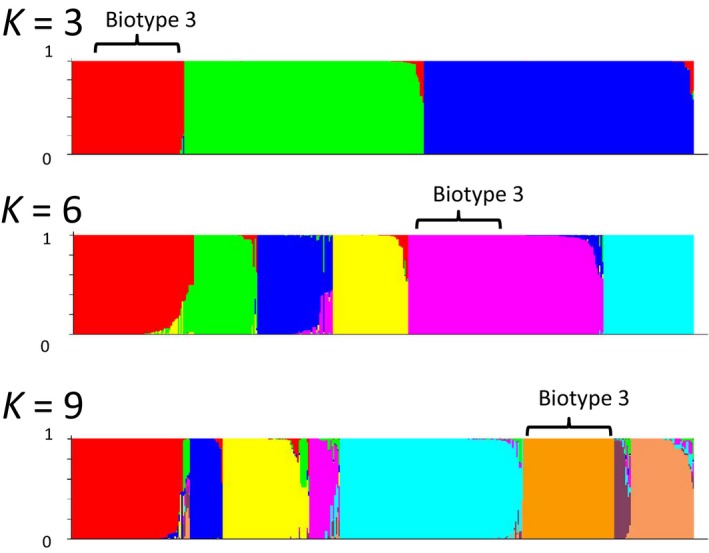
Inference of genetic clusters for 194 biotype 1 isolates. This assignment was implemented using STRUCTURE and based on 100 predefined isolates. K represents the number of given clusters. Each vertical bar represents an isolate, and the different colors represent the inferred clusters by the assignment analysis using STRUCTURE software. The mixture coloration of each bar indicates the probability with which an isolate can be assigned to a particular cluster

There are 294 isolates analyzed in STRUCTURE. Among them, 100 isolates were used as training datasets. One hundred and ninety‐four isolates were assigned into predefined clusters. The number of predefined clusters ranged from 3 to 9 (Figure 3). The sample sizes for each of the nine categories are listed in Table 6. In Figure 3, each colored vertical line represents an isolate. Isolates were assigned into potential attributed clusters, and different clusters are shown by different colors. For the hybrid vertical line (isolates), the estimated probability of the origin is shown by different colors. No matter how many clusters are specified, the biotype 3 isolates are always grouped together and distinguished from the other groups. This observation indicates that the most important feature of the *V. vulnificus* phylogeny is the molecular divergence of biotype 3 isolates.

**Table 6:** Number of isolates included from each super‐region for each of three sources

Region and source	Isolate number
Asia, Environment	4
Asia, Human	33
Asia, Aquatic sources	69
Europe, Environment	95
Europe, Human	14
Europe, Aquatic animals	22
USA, Environment	3
USA, Human	29
USA, Aquatic animals	25
Total	294

## 4 DISCUSSION

Utilizing phylogenetic tools, we adduced four findings in this study. First, a significant geographical variance of *V. vulnificus* was identified for the first time. Second, this geographical variance is more phylogenetically important than the host associations of *V. vulnificus*. Third, the classification of *V. vulnificus* is hierarchical. Biotype 3 has the most divergence from the other biotypes, which is then followed by the geographical region, then the host association. Fourth, this study developed an analytical strategy to identify the geographical effects of genotypic variance, and this strategy can be widely applied to other bacteria.

Geographical locations and different host sources can create genotype diversity. In the current analyses, host attribution was the least useful feature for the classification of *V. vulnificus*. The separation of biotypes 1 and 2 also provided little information as to how to classify it. These findings can partially explain the unsuccessful attempts at classifying *V. vulnificus* according to host (human vs. environment). One possible reason why most previous attempts focused solely on host attributions, which have proven nondefinitive, is technical sampling limitations (Levin, 2005; Wong et al., 2005).

All of the *V. vulnificus* biotype 3 isolates were sampled from Israel, and they were the cause of multiple outbreaks in Israel (Bisharat et al., 1999; Paz, Bisharat, Paz, Kidar, & Cohen, 2007). Biotype 3 may be the recombination product of biotypes 1 and 2 (Bisharat et al., 2005). The BEAST analyses and STRUCTURE analyses demonstrated that biotype 3 has emerged recently. In general, the classification of *V. vulnificus* only based on the isolates biotype 1 and biotype 2 is indistinguishable. However, biotype 3 isolates are significantly distinguishable from other isolates.

Previous classifications based solely on environmental and clinical sources have proven unsuccessful for distinguishing avirulent and virulent strains. The reasons for this unsuccessful classification are largely due to two factors. The first is sampling bias. In the current MLST database (as of Jan 1, 2017) of *V. vulnificus*, there are 452 isolates in total, and among them, 105 isolates are from the environment and 170 from clinics. For unique STs, there are 319 unique STs in the current MLST database. Among them, there are 100 STs from the environment and 83 isolates from humans. This could be a reflection of the diversity of sequence types in their ecology. The second factor is the unequal uptake rate of *V. vulnificus* into the human food chain between environmental and clinical strains. The human sources of *V. vulnificus* are mainly specific seafood, such as oysters or eels. Previous studies have shown that the survival rate of environmental strains is lower than clinical strains in the bodies of oysters (Froelich & Noble, 2014; Froelich, Ringwood, Sokolova, & Oliver, 2010). Therefore, the chances of clinical strains entering the human food chain are much higher than those of the environmental strains. Despite the existence of sampling bias and an unequal uptake rate, neither of these factors means that environmental strains are not virulent. The potential sampling bias is also consistent with the proven potential virulence of environmental strains (Amaro et al., 1994; Starks et al., 2000). Furthermore, this classification does not reflect climate effects. Climate factors in different geographical locations can be treated as snapshots of the time at which climate changes took place. Many advanced computational and statistical models will be necessary to apply climate factors to evolutionary and ecological analyses of bacteria in future work.

For neighbor‐joining analyses (Figure 1a), there are European clusters and Asian cluster of analyzed isolates. Within the main cluster of European isolates, there are in total of 15 isolates from Asia, and 34 isolates from United States. Within the Asian cluster, there are 12 isolates from Europe and 12 isolates from United States. These results are approximately consistent with the assignment from STRUCTURE analyses (Table 5). For US isolates, there is no apparent US cluster. The potential reasons could be due to the effect of human activity, such as travel, trade, pollution concentration, or ocean fishing.

Both the STRUCTURE and NJ tree results (Figures 1 and 3) demonstrate that biotype 3 has the most apparent separation between *V. vulnificus* types, regardless of the sampling source. Although it has only been reported in Israel so far, this sequence type caused two severe outbreaks within a 10‐year span (Bisharat et al., 1999; Paz et al., 2007), and thus, it represents a recently evolved and highly virulent group. The clear separation of biotypes indicated that they were more important than either geographical sampling location or the host source, although STRUCTURE analysis also revealed that the latter two both play a role in the evolution and genotype diversity of *V. vulnificus*. The geographical variation of *V. vulnificus* genotypes exceeds the host association, a finding that is also supported by the AMOVA results.

In summary, biotype 3 defines the first divergence level, which is more important than geographical location, and the effect of geographical location exceeds that of the source attribution. In other words, the classification of *V. vulnificus* should be considered as hierarchical rather than one dimensional. This is one of the first studies to successfully detect geographical effects on the ecology and evolution of *V. vulnificus*. The findings presented here provide strong evidence that the evolution of bacteria is affected by their micro‐ and macro‐ecology.

Further efforts should be made to collect genomic data from *V. vulnificus* and related global climate data. Even with the currently limited number of isolates, we can still detect significant effects; more data would allow for more precise analyses. Future research should focus on integrating advanced tools from interdisciplinary fields, including statistics, phylogenetics, computer science, and microbiology. Although it is just a starting point, we have demonstrated the importance of integrating geographical factors into the evolutionary analysis of bacteria.

## DATA ARCHIVING STATEMENT

The datasets generated during the current study have been deposited in the public available repository, https://dataverse.harvard.edu/dataset.xhtml?persistentId=doi:10.7910/DVN/FFKIMS.

## CONFLICT OF INTEREST

The author declares no conflict of interest.
