# Supplementary material for: Uncovering the geographical and host impacts on the classification of Vibrio vulnificus
Source: Evol Appl. 2018 Feb 20;11(6):883–90. doi: 10.1111/eva.12602 (PMC5999204; doi:10.1111/eva.12602)
Supplement: Supplementary file 1 [file EVA-11-883-s001.docx]

**Supplementary table1.** Number of assigned isolates included from each super-region for each of three sources.

| Region | Source | Isolate number |
| --- | --- | --- |
| Asia | Environment | 6 |
| Asia | Human | 60 |
| Asia | Aquatic animals | 116 |
| Europe | Environment | 96 |
| Europe | Human | 81 |
| Europe | Aquatic animals | 36 |
| USA | Environment | 3 |
| USA | Human | 29 |
| USA | Aquatic animals | 25 |

**Supplementary table2.** Number of predefined isolates included from each super-region for each of three sources.

| Region and source | Isolate number |
| --- | --- |
| Asia, Environment | 2 |
| Asia, Human | 14 |
| Asia, Aquatic animals | 24 |
| Europe, Environment | 26 |
| Europe, Human | 8 |
| Europe, Aquatic animals | 8 |
| USA, Environment | 2 |
| USA, Human | 11 |
| USA, Aquatic animals | 5 |
| **Total** | **100** |
